# EEGLAB, SIFT, NFT, BCILAB, and ERICA: New Tools for Advanced EEG Processing

**DOI:** 10.1155/2011/130714

**Published:** 2011-05-05

**Authors:** Arnaud Delorme, Tim Mullen, Christian Kothe, Zeynep Akalin Acar, Nima Bigdely-Shamlo, Andrey Vankov, Scott Makeig

**Affiliations:** ^1^Swartz Center for Computational Neuroscience, Institute for Neural Computation, University of California San Diego, La Jolla, 92093 CA, USA; ^2^Université de Toulouse, UPS, Centre de Recherche Cerveau et Cognition, 31062 Toulouse, France; ^3^CNRS, CerCo, 31062 Toulouse, France; ^4^Department of Cognitive Science, University of California San Diego, La Jolla, 92093 CA, USA; ^5^Department of Neurosciences, School of Medicine, University of California San Diego, La Jolla, 92093 CA, USA

## Abstract

We describe a set of complementary EEG data collection and processing tools recently developed at the Swartz Center for Computational Neuroscience (SCCN) that connect to and extend the EEGLAB software environment, a freely available and readily extensible processing environment running under Matlab. The new tools include (1) a new and flexible EEGLAB STUDY design facility for framing and performing statistical analyses on data from multiple subjects; (2) a neuroelectromagnetic forward head modeling toolbox (NFT) for building realistic electrical head models from available data; (3) a source information flow toolbox (SIFT) for modeling ongoing or event-related effective connectivity between cortical areas; (4) a BCILAB toolbox for building online brain-computer interface (BCI) models from available data, and (5) an experimental real-time interactive control and analysis (ERICA) environment for real-time production and coordination of interactive, multimodal experiments.

## 1. Introduction

A variety of new signal processing methods have been applied to EEG signal processing over the past fifteen years [1]. These new methods require new tools to allow routine processing of EEG data, and also make possible the analysis of multimodal data collected using more complex experimental designs than previous analysis methods allowed. Here we summarize a collection of new tools designed to be made freely available for nonprofit use and which integrate with the well-established EEGLAB software environment [2], an interactive, graphic interface menu and command line script-based environment for processing electrophysiological data. Since its introduction in 2001, EEGLAB has become a widely used platform for processing of biophysical data and for sharing of new signal processing approaches. Recently, we have introduced a number of new EEGLAB-associated toolboxes: NFT, a neuroelectromagnetic forward head modeling toolbox [3] is a new toolbox for electrical head modeling, an essential first step in electrophysiological source localization. SIFT, a source information flow toolbox, allows users to apply a wide range of recently published methods for assessing effective connectivity between EEG signals including quasi-independent sources of EEG activity. Finally, the ERICA framework, composed of the Datariver, Matriver, and Producer toolboxes, and the interoperable BCILAB toolbox manage real-time synchronization and online processing of EEG and other multimodal data streams. ERICA also handles feedback and delivery of appropriate sensory stimuli to participant(s) and/or to a control system they are operating [4]. 

**Figure 1:**
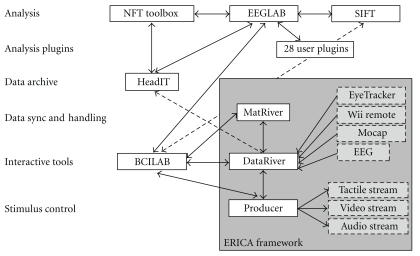
Complete electrophysiological experiment control, data collection, analysis, archiving, and meta-analysis suite: the EEGLAB environment for data analysis; the ERICA framework for data recording, online analysis, and stimulus control; the BCILAB toolbox for online and offline classification and BCI; the SIFT toolbox for information flow modeling; HeadIT, an archival data and tools resource under development for laboratory or archival data storage, retrieval and meta-analysis; dashed lines indicates planned interfaces under construction.

Figure 1 depicts how the new toolboxes interact and may connect to a distributed data archiving environment (here, the proposed HeadIT data and tools resource [5]).  Table 1 lists the components of the Swartz Center for Computational Neuroscience (SCCN) software suite. Note that we designate by “EEGLAB plug-in” any function, toolkit, or more organized and ambitious projects such as fully operational and standalone toolboxes or signal processing toolboxes that use the EEGLAB data structure and conventions. In this paper, we designate by “framework” any grouping of tools or toolboxes in which common code providing generic functionality can be selectively overridden or specialized by user code to provide custom functionality. For instance the ERICA is a framework centered around the concept of a “Data River” and including the clients and server implementing this concept.

## 2. EEGLAB

EEGLAB is an interactive menu-based and scripting software for processing electrophysiological data based under the Matlab interpreted programming script environment [2]. EEGLAB provides an interactive graphical user interface allowing users to flexibly and interactively process their high-density electrophysiological data (of up to several hundreds of channels) and/or other dynamic brain time series data. EEGLAB implements common methods of electroencephalographic data analysis including independent component analysis (ICA) and time/frequency analysis. EEGLAB has become a widely used platform for applying and sharing new techniques for biophysical signal processing. At least 28 plug-ins have been implemented and released by user groups. Here we describe recent developments in EEG software interoperative with EEGLAB. Several of the new tools are Matlab applications that conveniently plug in to the EEGLAB menu (or may also be run as stand-alone applications). 

Key EEGLAB features include

1. an event structure and functions for importing, editing, and manipulating event information. Users can select (sub)epochs time-locked to classes of events and can sort trials for visualization based on values in any event field (e.g., subjects' reaction time),
2. independent component analysis (ICA) decomposition of electroencephalographic data [6]. Though ICA data analysis methods have now been incorporated into most commercial software processing EEG data (BrainVision, Neuroscan, BESA), EEGLAB has the most extensive repertoire of processing and data evaluation tools for ICA-based data analysis,
3. ready adaptability to users with different levels of programming sophistication. EEGLAB unique “history” features build scripts as users navigate through menus, allowing users to “replay”, vary, or extend their data processing through easily constructed Matlab scripts. Users can either interact only with the EEGLAB graphic interface, call EEGLAB functions directly from the Matlab command line, or write their own Matlab scripts using modular EEGLAB functions and documented data structures,
4. a truly open source philosophy, allowing any researcher to build and distribute plug-in functions or toolboxes that appear automatically in the EEGLAB menu windows of their users. This structure ensures stability of core code that a handful of expert users modify while, at the same time, allows easy inclusion of new algorithms and methods by other users.

EEGLAB comprises more than 400 Matlab functions totaling more than 50,000 lines of programming. First developed under Matlab v5.3 on Linux, EEGLAB currently runs under all versions of Matlab v7 running on Linux, Unix, Windows, and Mac OSX. Since the Matlab program is not free itself, we have also used the Matlab compiler to compile EEGLAB for those users who do not have access to Matlab. To our knowledge, 28 user-initiated EEGLAB plug-ins have been developed and made available. The online EEGLAB tutorial comprises more than 300 pages of documentation. In addition, each of the 400 stand-alone modular EEGLAB functions contains its own documentation. EEGLAB has been downloaded more than 65,000 times from 88 country domains since 2003. As of April 2010, 9,218 unique opt-in users are currently on the EEGLAB mailing lists.

## 3. The EEGLAB STUDY.Design Framework

 The EEGLAB STUDY.design concept was introduced in June, 2010 in EEGLAB v9. Complex event-related experiments typically include a number of different types of events. Statistical contrasts between EEG activities time locked to different subsets of these event types require researchers to be able to define custom sets of independent variables for different statistical treatments of the same data. The new STUDY.design framework in EEGLAB allows users to freely define independent and dependent variables and to analyze data channel or independent component (IC) activities across subjects using mean event-related potential (ERP), power spectrum, event-related spectral perturbation (ERSP) [7], and intertrial coherence (ITC) [8] measures for any number of sets of event-related data trials time locked to different sets of events, each set of trials termed a STUDY “condition”.

**Figure 2:**
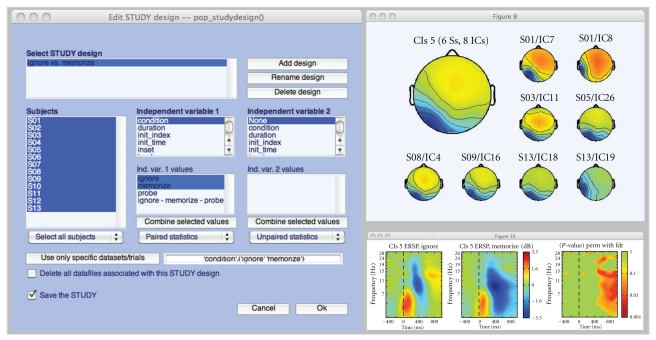
EEGLAB STUDY design interface using the tutorial STUDY data available via the EEGLAB wiki (http://sccn.ucsd.edu/wiki/eeglab). The three push buttons at the top may be used to add a new design (“Add design”), rename a design (“Rename design”), or delete a design (“Delete design”). The “Independent variable 1” list helps define independent variables. The list of independent variables is automatically generated based on the STUDY definition information and individual data set event types. For a given independent variable, it is also possible to select a subset of its values or to combine some of its values. For instance, in this example the user has selected “ignore” and “memorize” stimuli as values for the independent variable “condition”. The “Subject” list contains the subjects to include in a specific design. Unselecting a given subject from the list excludes him/her from further data analysis within the design. Once a design is selected, measures including ERPs, mean spectra or event-related spectral perturbations (ERSP) may be plotted. Here, we have plotted the event-related spectral perturbations of an independent component (IC) cluster in the selected STUDY.design. In the top right panel, the scalp maps of one IC cluster are shown—the large map representing the average scalp map. In the bottom right panel, mean cluster ERSPs are shown for Ignore versus Memorize letter trials, and their significant differences are assessed using permutation-based statistics and a false discovery rate method to correct for multiple comparisons.

For example, a STUDY might contain data sets for two conditions from two groups of subjects (a 2 × 2 (condition, group) statistical design). Statistical comparisons might be targeted to look at main effects and interactions of condition and group in this design, or at contrasts between selected (1 × 2) pairs of conditions or groups.  Figure 2 shows the EEGLAB STUDY.design graphic interface by means of which users can create new designs and select independent variables to include in them.

Building a STUDY design involves multiple steps. Users begin by preprocessing binary EEG data files generated by proprietary EEG recording software; for each subject, this involves importing raw data, re-referencing, filtering and removing artifacts. Once these data sets have been pre-processed, users then have to import the subject data sets into a STUDY. Creating a STUDY design for analysis then allows statistical group comparison of data measures for different conditions (e.g., time locked to specific event types) for each subject. For example, in an oddball paradigm comprised of trials time locked to target, distractor, and standard stimuli, users might want to contrast these three types of trials using a 3 × 1 design. Alternatively, they might want to contrast distractor and target stimulus-locked trials, considered together, with responses to standard stimuli. The STUDY design feature of EEGLAB allows users to easily investigate such contrasts. In a STUDY with N subject groups, the STUDY design scheme also allows users to look at group effects for each condition using a 2 × *N* design. 

All of the above design concepts may be implemented within a single STUDY using multiple STUDY.design specifications. Finally, use of multiple designs may also be useful for testing different signal processing options. For instance, one might create two identical STUDY designs, one computing time/frequency measures using fast fourier transforms (FFT) and the other using wavelets. Once computed, the user can toggle between designs to compare results using the two types of time/frequency decomposition.

EEGLAB uses statistical tools including surrogate and parametric statistics to perform hypothesis testing on STUDY designs. Surrogate tests involve bootstrap or permutation methods. Depending on the design type, statistical hypothesis testing using *t*-test, one-way ANOVA or two-way ANOVA—or their surrogate-data equivalents—are performed for paired data or unpaired data designs. Finally, the False Discovery Rate (FDR) algorithm is applied to correct for multiple comparisons [9]. Using these simple yet powerful statistical tools, EEGLAB allows comparison of multiple experimental designs applied to a given data STUDY.

When working with data from multiple subjects using the STUDY design framework, users may analyse either IC, scalp channel, or other types of component activities associated with individual subjects. Decomposition of the data into ICs allows inclusion of source localization information, since many ICs strongly resemble the projection of a single equivalent current dipole, presumably reflecting their origin in a single locally synchronized cortical patch. The neuroelectromagnetic forward head modeling toolbox (NFT) thus allows for more precise source localization of IC processes for each subject using subject-adapted forward electrical head models.

## 4. The Neuroelectromagnetic Forward Head Modeling Toolbox (NFT)

Our previous work has shown that some ICA component scalp topographies are highly compatible with compact cortical domains of local field synchrony that may be localized in the brain [1, 10, 11] using a four-shell spherical model or the standard boundary element method (BEM) head model included in the EEGLAB Dipfit plug-in (http://sccn.ucsd.edu/wiki/A08:_DIPFIT). When additional subject information is available, more precise localization approaches are possible. To obtain accurate source localization one needs to use a realistic electrical head model that reflects the actual electrical and geometric properties of the head. NFT adds a realistic head modeling framework to the spherical and MNI head models already provided by Dipfit within EEGLAB. The NFT framework automates most of the tasks needed to generate a realistic head model from magnetic resonance (MR) images and/or from measured EEG sensor coordinates, and provides advanced boundary element method (BEM) and Finite Element Method (FEM) solvers for estimating the projected scalp fields for a given set of possible brain source areas, thus estimating solutions to the “forward” EEG modeling problem [3].

NFT is accessible from the EEGLAB graphic interface as an EEGLAB plug-in. The toolbox provides both a Matlab command line and graphical user interface for generating realistic head models from available subject information, and for solving the forward problem numerically to provide a lead-field-matrix for a given source space and sensor distribution. This makes it easy to integrate a forward head model produced by NFT into any inverse source localization approach.

**Figure 3:**
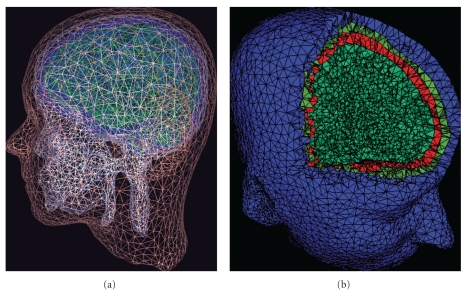
Two examples of (a) a set of subject head BEM meshes (modeling scalp, skull, cerebrospinal fluid (CSF), and cortex tissue boundaries) and (b) a FEM head volume for the same subject with 3-D voxels for scalp, skull, and brain tissues shown in different colors.

NFT performs the following steps:

1. Segmentation of MR images: If a 3-D whole-head structural T-1 MR image of the subject's head is available, the toolbox can segment the scalp, skull, CSF, and brain tissues.
2. High-quality head models: The accuracy of numerical solutions to an inverse source localization problem depends on the quality of the underlying meshes that model conductance changes at tissue boundaries. NFT can create high-quality surface meshes from segmented MR images for use in BEM head model. FEM meshes may be generated from the BEM surface meshes using the open source Tetgen tool [12]. Two examples of FEM and BEM meshes generated using NFT are shown in Figure 3.
3. Warping a template head model: While use of a subject whole-head MR image is the preferred way to generate a realistic head model, such an image may not always be available. NFT can generate a semirealistic head model of the subjects' head by warping a standard template head model to the digitized 3-D electrode coordinates, when these are available.
4. Coregistration of electrode positions with the head mesh: NFT has a two-step (manual and automatic) coregistration function for aligning the digitized electrode locations to the scalp mesh.
5. Accurate and efficient forward problem solution: The NFT uses high-performance BEM and FEM implementations from the open source METU-FP Toolkit (http://www.eee.metu.edu.tr/metu-fp) [13, 14] for bioelectromagnetic field computations.

We have successfully used NFT to model realistic cortical source spaces comprising a large number of dipolar elements that we assume are oriented perpendicular to the local cortical surface which was extracted from subject MR head images using tessellated FreeSurfer gray and white matter surfaces [15]. We created a multiscale cortical patch basis on this surface by selecting seed points (single voxel dipoles), then extended each patch conformally to a set of Gaussian-tapered patches with areas in the range ~50–200 mm^2^ [16]. NFT thus may allow precise source localization of IC processes based on accurately modeled electrical current flow consistent with the individual subject head anatomy.

FEM modeling is a recent addition to NFT (NFT 2.0) and patch-based source space generation will be integrated into NFT in 2011. In the future, the NFT model will also be able to incorporate models of current anisotropy based on white-matter distribution information extracted from diffusion tensor/weighted imaging (DTI/DWI) head images co-registered with structural MR images.

## 5. Analyzing Source Information Flow Dynamics Using SIFT

Once activity in specific brain areas have been identified using source separation (e.g., ICA), and localized (e.g., using NFT), it is possible to look for transient changes in the independence of these different brain source processes. Advanced methods for noninvasively detecting and modeling distributed network events contained in high-density scalp EEG data are desirable for basic and clinical studies of distributed brain activity supporting behavior and experience. In recent years, Granger Causality (GC) and its extensions have increasingly been used to explore “effective” connectivity (directed information flow, or causality) in the brain based primarily on observed ongoing or event-related relationships between channel waveforms. While many landmark studies have applied GC to invasively recorded local field potentials and spike trains, a growing number of studies have successfully applied GC to noninvasively recorded human EEG and MEG data (as reviewed by Bressler and Seth [17]). 

Based on the prediction error of autoregressive (AR) models, a process (A) is said to *Granger-cause *another process (B) if past values of process A, in addition to past values of process B, help to linearly predict future values of process B beyond what can be achieved by using past values of process B alone [18]. Using multivariate autoregressive (MVAR also referred to in the literature as VAR or MAR) models, the GC concept has been extended to an arbitrary number of signals, which may include a collection of source activities in the brain. Using this approach, through Fourier-transformation of the MVAR coefficient matrices, we can obtain the transfer and spectral density matrices (power), and ordinary, multiple, and partial coherences, where the latter quantity expresses the amount of phase coherence between two channels after subtracting out the part of the interaction which can be explained by a linear combination of all other channels. From these quantities, we can derive a frequency-domain representation of bivariate GC as well as several frequency-domain measures of directed conditional (multivariate) dependence closely related to Granger's definition of causality such as the (direct) directed transfer function (dDTF, DTF) and partial directed coherence (PDC). These and related estimators describe different aspects of network dynamics and thus comprise a complementary set of tools for MVAR-based connectivity analysis within the well-established and interpretable framework of GC [19]. To study transient causal dynamics of nonstationary phenomena, adaptive MVAR (AMVAR) approaches may be applied using locally-stationary sliding windows [20], Kalman filtering, or spectral matrix factorization. These approaches can be used to explore finely-resolved time- and frequency-dependent dynamics of directed information flow or causality between neuronal sources during cognitive information processing. Baseline significance levels for causal influence are typically obtained by a modification of a surrogate “phase randomization” algorithm [21]. This and other bootstrap, permutation, and analytical tests can be used to establish rigorous confidence intervals on estimated connectivity. Additional details on all aforementioned methods can be found in [19].

SIFT is a toolbox for modeling and visualizing information flow between sources of EEG data, possibly after separating the data into (instantaneously) maximally independent processes using ICA. The toolbox currently consists of four modules, (1) data preprocessing, (2) model fitting and connectivity estimation, (3) statistical analysis, and (4) visualization. The first module contains routines for normalization, downsampling, detrending, and other standard preprocessing steps. The second module currently includes support for several adaptive MVAR modeling approaches. From the fitted model, the user can chose to estimate spectral power, coherence, and frequency-domain connectivity, selecting from over fifteen measures published to date. The third module includes routines for surrogate statistics (phase-randomization and bootstrap statistics) for all measures, and analytic statistics for partial directed coherence and directed transfer function measures. The fourth module contains novel routines for interactive visualization of information flow dynamics and graph-theoretic measures across time, frequency, and anatomical source location. A graphical user interface allows easy access to the SIFT data processing pipeline.

**Figure 4:**
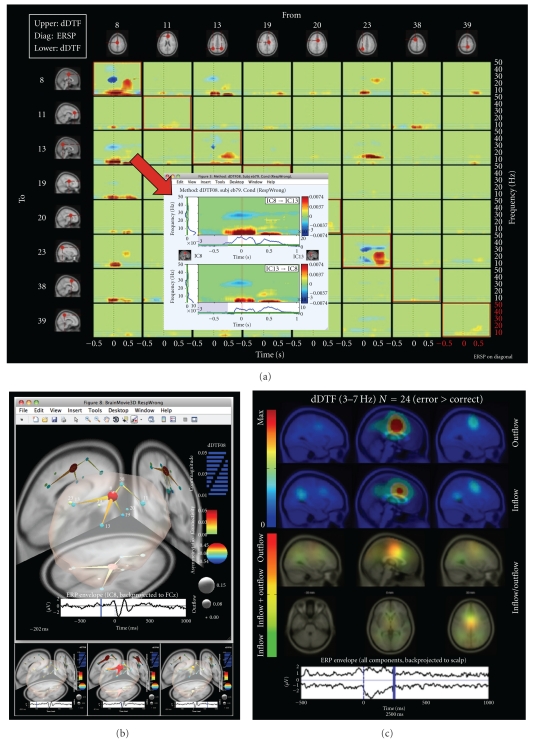
EEG-based brain connectivity analysis and visualization using SIFT. (a) An interactive time-frequency grid demonstrating transient bursts of theta (3–7 Hz) and delta (1–3 Hz) band information flow during error commission, estimated using the direct directed transfer function (dDTF), between a subset of independent component (IC) sources. Dashed vertical line denotes time of erroneous button press. Callout shows an expanded view of information flow to/from sources 8 and 13, obtained by clicking on the respective grid cell. (b) Several frames from an interactive BrainMovie3D animation showing an event-related causal relationship in the theta band between these sources (200 ms (top) and −520, 40, and 600 ms (bottom) relative to an erroneous button press). Ball (node) color and size denotes asymmetry ratio (red: causal source, blue: causal sink) and outflow strength, respectively, for that IC. Cylinder (edge) color and size denote connectivity strength. The event-related potential of IC8 (red, medial), back-projected to a superior electrode is superimposed below each frame (blue bar denotes frame index). This shows a network interpretation of the classic “error-related negativity” (ERN) phenomenon observed during error-processing. (c) A frame from a causal projection movie showing mean net causal inflow (green) and causal outflow (red) in the theta band at each brain location during error commission across 24 subjects. Note the significant causal outflow from or near anterior cingulate cortex, thought to be critically involved in error-processing, during and following the negative peak of the ERN.

A key aspect of SIFT is that it focuses on estimating and visualizing multivariate effective connectivity in the source domain rather than between scalp electrode signals. This should allow us to achieve finer spatial localization of the network components while minimizing the challenging signal processing confounds produced by broad volume conduction from cortical sources (as well as nonbrain sources) to the scalp electrodes. SIFT may help find transient, dynamic network events that link spatially static component processes (Figure 4). The toolbox may also be used for effective connectivity analysis and visualization of phenomena in electrocorticographic (ECoG) data, for example, to identify sources and directions of information flow at onsets of and during epileptic seizures. 

While the first test release of SIFT contains a number of popular MVAR-based effective connectivity measures, we are working on incorporating additional phase-amplitude coupling and transfer entropy measures. In the EEGLAB tradition, the architecture of the toolbox is also designed to allow easy addition of new methods from the user community. Another group analysis module, in development, will also be included in the upcoming second test release. This will afford clustering-based and Bayesian techniques for obtaining estimates of source-domain connectivity with confidence intervals over a subject population. The analysis framework described above allows exploration of EEG source-domain connectivity following the use of EEGLAB and NFT routines for ICA-based source separation and localization. We are currently evaluating the relative suitability of different source separation algorithms when combined with MVAR-based connectivity algorithms, and will further develop the toolbox accordingly. In the near future, we plan to interface SIFT with the BCILAB toolbox, discussed below, with the hope of applying these methods online in advanced brain-machine interfaces for real-time EEG processing, cognitive monitoring, and feedback applications.

## 6. The Experimental Real-Time Interactive Control and Analysis (ERICA) Framework

For the purpose of real-time data acquisition and processing, we have developed an online EEG and multimodal data collection, processing, and interactive feedback environment, ERICA. Processing of EEG data in real-time software applications requires, first, organized handling of data controlling its streaming into online data processing (e.g., data-adaptive BCI or other feedback) routines whose outputs, combined into synchronized data streams (figuratively a “data river”), can be used to control or adapt ongoing stimulation processes. Synchronization of different asynchronous streams in real time over a local network may prove difficult; the originality of the ERICA framework comes from solving these issues in an efficient and elegant manner.

The ERICA framework is based on a unique streaming data management and real-time cross-platform synchronization application called DataRiver developed from an ADAPT data acquisition and stimulation control environment [22]. The Producer software is a DataRiver client that controls stimulus presentation in a flexible way using Variéte, an original scripting language. MatRiver, another DataRiver client, allows direct read/write access to DataRiver data streams from within Matlab processes.

The central application driving development of ERICA is the development of mobile brain/body imaging (MoBI) data acquisition and analysis methods [23]—the simultaneous study of what the brain is doing (assessed via distributed EEG source dynamics), what the brain is sensing (via audiovisual scene recording), and what the brain is controlling (the totality of our behavior assessed by body motion capture, eye tracking, etc.) in performing naturally motivated actions in ordinary 3-D task environments.

To allow real-time analysis, data streams acquired by separate devices first need to be synchronized. Such streams are, by definition, asynchronous, even when they are acquired at the same nominal sampling frequency because independent clocks are used for data acquisition in each device. In addition, the sampling rates for different data sources may differ significantly: while EEG is usually sampled between 250 Hz and 2,000 Hz, video, body motion capture or subject behavioral responses may be acquired at a much lower sampling rate, and audio data streams at still higher sampling rates. For synchronization purposes, another important challenge is dealing with sporadic delays introduced by equipment acquisition, network, and operating system buffers that ensure overall regularity of data samples at the cost of ms-level time delays. For data acquired through an IP socket connection, network delays may be significant and constantly varying. Finally, Windows or any other multitasking operating system introduces variable delays in the processing of asynchronous flows—in a multitasking system, data are most often processed only when the corresponding task or program is activated and not when the data first becomes available. 

DataRiver was developed in an attempt to solve these synchronization problems. DataRiver is a flexible and universal high-precision synchronization engine, providing a strong and near real-time synchronization of simultaneous data streams. It has been designed and tested with accuracy of better than 2 ms, even when synchronizing data acquisition streams from different computers (running Windows, Unix, Linux, or Mac OSX) over a local area network or the internet subnet. The DataRiver application interfaces several hardware and is typically seen as a server to DataRiver Clients that display or process data. However, each DataRiver client can also add output data to the “data river,” so the strict concept of server and client does not apply.

**Figure 5:**
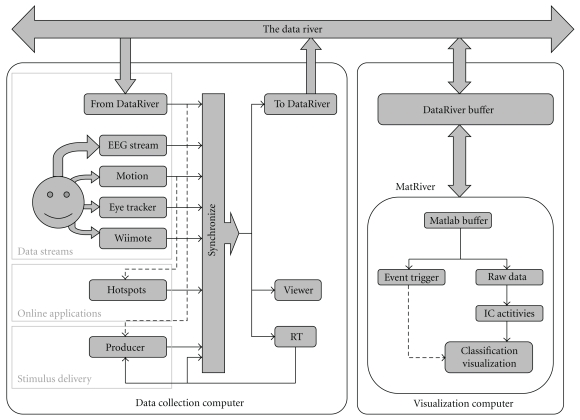
An ERICA data flow involving two separate computers each running an instance of the DataRiver application. Dashed lines indicate control signals. Here, computer visualization is performed using the Matlab DataRiver client MatRiver.

The flexibility of the ERICA framework stems from its modular design—data output from a variety of devices are managed by specialized device drivers that convert each data stream into a device-independent stream. These streams are then merged in real time and combined into a “river” (hence the name DataRiver). DataRiver device drivers are currently available for several types of input devices and data systems including Biosemi EEG, PhaseSpace and OptiTrack motion capture systems, eye trackers, and the Wii remote (Nintendo, Inc.). This enables the rapid development of a wide range of experimental paradigms that can be tailored for a variety of multimodal experimental or application environments. Data from incoming DataRiver data streams may be used in real time by clients for recording, online data processing, and/or to provide feedback to the subject(s) being monitored. DataRiver has integrated support for data exchange in real time between one or more remote computers connected to a local area network (LAN), enabling distributed and cooperative experiments (Figure 5). New drivers and online data processing applications can easily be added to DataRiver to meet evolving research needs.

MatRiver is a MATLAB DataRiver client optimized for real-time EEG data processing, buffering and visualization using the OpenGL-based Simulink 3-D toolbox (The MathWorks, Inc.). MatRiver communicates with DataRiver by calling a binary library of functions under Windows OS. MatRiver allows online performance of common EEG preprocessing steps such as channel selection, channel re-referencing, frequency filtering and linear spatial filtering using a pre-defined ICA source signal unmixing matrix [6]. Most often, these steps may be accomplished in near real time by directly calling relevant EEGLAB functions. MatRiver also includes routines to dynamically detect “bad” channels and compensate for them by taking into account a linear ICA source propagation model. Preprocessed channel or independent component (IC) signals are accumulated and can subsequently be used for classification using MATLAB tools such as BCILAB (see following). MatRiver uses Matlab “timers” to run in the background allowing real-time processing in a nonblocking manner, even including near real-time interactive exploration of the incoming data from the Matlab command line. Continuous visualizations of data characteristics such as alpha band energy are also possible. In short, Matriver functions provide an elegant and straightforward pipeline for EEG preprocessing and classification using the rich tool set and programming simplicity of MATLAB.

## 7. Designing Brain-Computer Interfaces with BCILAB

 After results of data stream synchronization and preprocessing have been accomplished within the ERICA framework, one may use BCILAB, an open-source MATLAB toolbox and EEGLAB [2] plug-in, to support brain-computer interface (BCI) research, and more generally, the design, learning (or adaptation), use, and evaluation of real-time predictive models operating on signals. The main objects of study in BCILAB are Brain-Computer Interface (BCI) models [24], generally defined as systems that take human bio-signals as input and output estimates of some aspect of the subject's cognitive state. The signals processed by BCIs are traditionally restricted to EEG signals, but may include other modalities, such as motion-capture data or skin conductance (plus context parameters such as vehicle state, previous events, etc.). These data can be processed either using BCILAB running as a data processing node in a real-time experimentation environment (e.g., ERICA), or offline simulated real-time applications to existing data. The classifier outputs of a BCI can be streamed to a real-time application to effect stimulus or prosthetic control, or may be derived post hoc from recorded data, for example for statistical analysis of the model's prediction accuracy when applied to a database of previously recorded data. BCILAB is highly flexible and most accessible cognitive states can be investigated, for example imagined movements (affecting in sensorimotor mu rhythms), surprise (provoking, e.g., the oddball P3), or indicators of drowsiness.

The tools provided by BCILAB facilitate most steps in BCI research, including the design, implementation, learning, evaluation, and on- or off-line application of BCI (or other) models. Further tasks, including the exploration of recorded data and visualization of model parameters may be supported using EEGLAB tools. BCILAB has several layers, the top layer including a graphic interface, a scripting interface, and a real-time application interface, with a second layer including core model learning, model execution, and model evaluation functions. These core facilities in turn rely on a framework of “BCI paradigms”, which can be understood as prototypical template-like approaches to designing a BCI model. Pre-defined paradigms include common spatial patterns (CSP), logarithmic band-power estimates, and the approach proposed in the dual augmented lagrange framework [25]. A BCI “paradigm” defines the entire approach as it would be described in a publication, from raw input data to final output, and usually involves both a learning and a prediction stage, because sufficient performance can often only be achieved after a model is learned (or calibrated) based on sample data from a given session, subject, or task. BCI paradigms can be fully customized by the user, including removal or addition of entire components, but come with defaults for all their parameters, both to keep the learning curve gentle as well as to minimize the amount of information that must be specified.

At lower levels, BCILAB provides additional frameworks designed to be extensible and flexible and to have low implementation overhead. In particular, most BCI paradigms are defined within a “data flow” scheme wherein information is passed through several stages that are themselves plug-in frameworks: filters (signal processing), feature maps (feature extraction), and model learners as well as predictors/estimators (using machine learning). These frameworks are general enough to cover a wealth of implementations, such as adaptive/statistical epoched-signal processing, adaptive feature extraction, and classification/regression/density estimation, with general (discrete/continuous, multivariate, point-estimate/full-posterior) outputs. We are currently working to explore additional concepts including hierarchical Bayesian models spanning sessions, subjects and (related) tasks.

A simple use case of BCILAB is for the offline reanalysis of a BCI study. For example, given a collection of data sets, one per subject, containing imagined movements of either the left or the right hand in random order, with events “SL” and “SR” indicating the timing and type of the respective cue stimuli, a user of the Matlab-based BCILAB scripting interface may proceed as follows: For each subject,

1. Load a data set
2. ≫ eeg = io_loadset(‘session1.eeg');
3. Define an analysis approach (customizing parts of a standard paradigm)
4. ≫ approach =  {‘SpecCSP', ‘events',  {‘SL',‘SR'}, learner', ‘logreg'};
5. Apply the approach to the data, to get an estimate of its performance on the given data
6. ≫ [performance,model,statistics] = bci_train({‘data',eeg, …‘approach', approach});

This analysis gives the prediction accuracy results that are the key ingredient of most BCI publications (along with visualizations). Step (3) above also produces a calibrated predictive model which can be loaded into one of the provided real-time plug-ins (for ERICA, BCI2000 [48], and OpenViBE [49] real-time environments, with others forthcoming) for online testing. 

A major focus of the BCILAB toolbox is to allow, as much as possible, that competitive BCI estimation performance may be obtained using simply stated procedures (as above). For this purpose, a large collection of state-of-the-art methods have been provided and are listed in Table 2. A second, complementary focus is to provide rigorous analyses (e.g., for performance estimation) by default. For this purpose, a framework for automated cross-validation, systematic parameter search, and nested cross-validation is provided, and a suitable evaluation method is automatically chosen depending on the supplied data (though the evaluation method may also be customized). For example, if a single data set and at least one unknown parameter is provided by the user, nested block-wise cross-validation with safety margins is chosen by default. In a similar vein, to rule out common BCI research errors such as accidental non-causal signal processing, offline and online processing uses identical code.

BCILAB aims to be not just a collection of off-the-shelf tools to enable BCI experiments, but is designed to be a development platform for new BCI technology, facilitating the creation of new methods, approaches (e.g., combining existing methods), and paradigms. For this purpose, the toolbox provides extensive infrastructure, including, among others, the frameworks mentioned above, a small Mathematica-inspired symbolic expression system, an Adobe ASL-inspired declarative graphic interface property model, a decentralized distributed computing infrastructure (not dependent on MATLAB toolboxes), a generic dependency loader, a transparent multi-level cache for results, as well as bundled toolboxes for convenience. All BCILAB code is thoroughly documented, with additional citation-rich documentation for user-facing functions. Backwards compatibility to MATLAB 7.1 is attempted (and reached for most functionality except the graphic interface, which requires Matlab 2008a+, due to the use of objects).

## 8. Conclusion

The extended SCCN software suite centered on EEGLAB data structures and processing functions is an ongoing product of a coordinated effort to develop and test new methods for observing and modeling the dynamics of noninvasively observed electrophysiological activity in human cortex during a wide range of behavioral task performance, both *post hoc *and in real time. The tools we have developed towards this end include software for online data streaming and storage, advanced offline and online EEG analysis and prediction, source localization, and multivariate connectivity analysis and visualization. These build on and integrate with our well-established EEGLAB software suite that is now in use by thousands of researchers around the world. We plan to continue to extend and further coordinate these modular toolboxes with the hope that they will facilitate development of novel 21st century EEG analysis and data mining techniques which in turn will lead to transformative gains in our understanding of human neuroscience, cognition and behavior, facilitating a broad range of practical and clinical applications.

## Figures and Tables

**Table 1:** Components of the extended SCCN software suite.

Software	Since	Vers.	Licence	Open Src.	Platform	Web link
EEGLAB	2002	10.0	GNU GPL	Yes	Matlab	http://sccn.ucsd.edu/wiki/EEGLAB
NFT toolbox	2009	2.0	GNU GPL	Yes^†^	Matlab^†^	http://sccn.ucsd.edu/wiki/NFT
SIFT	2010	0.1a	GNU GPL	Yes	Matlab	http://sccn.ucsd.edu/wiki/SIFT
BCILAB	2010	0.9	GNU GPL	Yes	Matlab	http://sccn.ucsd.edu/wiki/BCILAB
ERICA	2009	1.0	Mixed*	Mixed*	Windows^††^	http://sccn.ucsd.edu/wiki/ERICA

*DataRiver, a central compiled C++ ERICA component, is free for noncommercial use. It is not open source.

^†^Contains a large number of precompiled C and C++ routines, all of them being open source.

^††^Many components also run under Linux and Mac OSX.

**Table 2:** Signal processing, feature extraction, and machine learning algorithms included in the BCILAB/EEGLAB framework.

Signal processing	Feature extraction	Machine learning algorithms
(i) Channel selection	(i) Multiwindow averages [26, 27]	(i) Linear discriminant Analysis (LDA) [28]
(ii) Resampling	(ii) Common Spatial Patterns (CSP) [29]	(ii) Quadratic discriminant analysis (QDA) [30]
(iii) Artifact rejection (spike detection, bad window detection, bad channel detection, local peak detection)	(iii) Spectrally-weighted common spatial patterns [31]	(iii) Regularized and analytically regularized LDA and QDA [30, 32]
(iv) Envelope extraction	(iv) Adaptive autoregressive modeling, from BioSig [33]	(iv) Linear SVM [34] (LIBLINEAR/CVX)
(v) Epoch extraction	(1) Dual-agumented lagrange (DAL) [25]	(v) Kernel SVM [34]
(1) Time-frequency window selection	(2) Frequency-domain DAL (FDAL)	(vi) Gaussian mixture models (GMM), 9 methods [35–37])
(2) Spectral transformation	(3) Independent Modulators [38]	(vii) Regularized and variational Bayesian logistic regression and sparse Bayesian logistic regression [39, 40]
(vi) Baseline filtering	(4) Multiband-CSP [41]	(1) Hierarchical kernel learning [42]
(vii) Resampling	(5) Multi-Model Independent component features	(viii) Relevance vector machines (RVM) [43]
(viii) Re-referencing		(1) group-sparse/rank-sparse linear and logistic regression [25]
(ix) Surface Laplacian filtering [44]		(2) high-dimensional Gaussian Bayes density estimator/classifier
(x) ICA methods (Infomax, FastICA, AMICA) [6, 45]		(3) Voting metalearner
(xi) Spectral filters (FIR, IIR)		
(xii) Spherical spline interpolation [46]		
(1) Signal normalization		
(2) Sparse signal reconstruction (NESTA, SBL [47], FOCUSS, l1; currently offline only)		
(3) Linear projection		
